# Impact of sunflower (*Helianthus annuus* L.) plastidial lipoyl synthases genes expression in glycerolipids composition of transgenic Arabidopsis plants

**DOI:** 10.1038/s41598-020-60686-z

**Published:** 2020-02-28

**Authors:** Raquel Martins-Noguerol, Antonio Javier Moreno-Pérez, Acket Sebastien, Manuel Adrián Troncoso-Ponce, Rafael Garcés, Brigitte Thomasset, Joaquín J. Salas, Enrique Martínez-Force

**Affiliations:** 1 Instituto de la Grasa-CSIC, Building 46, UPO Campus, Ctra. de Utrera km 1, 41013 Seville, Spain; 2 Alliance Sorbonne Universités, Université de Technologie de Compiègne, Génie Enzymatique et Cellulaire (GEC), UMR-CNRS 7025, CS 60319, 60203 Compiègne, Cedex France

**Keywords:** Molecular engineering in plants, Plant molecular biology, Plant physiology

## Abstract

Lipoyl synthases are key enzymes in lipoic acid biosynthesis, a co-factor of several enzyme complexes involved in central metabolism. Plant pyruvate dehydrogenase complex (PDH), located in mitochondria and plastids, catalyses the first step of fatty acid biosynthesis in these organelles. Among their different components, the E2 subunit requires the lipoic acid prosthetic group to be active. *De novo* lipoic acid biosynthesis is achieved by the successive action of two enzymes on octanoyl-ACP: octanoyltransferase (LIP2) and lipoyl synthase (LIP1). In this study, two plastidial lipoyl synthase genes from sunflower (*Helianthus annuus* L.) were identified (*HaLIP1p1* and *HaLIP1p2*), sequenced and cloned in a heterologous production system (*Escherichia coli*). Gene expression studies revealed similar expression patterns for both isoforms, with a slight predominance of *HaLIP1p1* in vegetative tissues and mature seeds. Tertiary structural models for these enzymes indicate they both have the same theoretical catalytic sites, using lipoyl-lys and 5-deoxyadenosine as docking substrates. The fatty acid profile of *E. coli* cells overexpressing *Ha*LIP1p1 and *Ha*LIP1p2 did not present major differences, and the *in vivo* activity of both proteins was confirmed by complementation of an *E. coli* JW0623 mutant in which lipoyl synthase is defective. Although no significant differences were detected in the total fatty acid composition of transgenic *Arabidopsis thaliana* seeds overexpressing any of both proteins, a lipidomic analysis revealed a redistribution of the glycerolipid species, accompanied with increased phosphatidylethanolamine (PE) content and a decrease in diacyglycerols (DAG) and phosphatidylcholine (PC). Depletion of the SAM co-factor caused by *Ha*LIP1p1 and *Ha*LIP1p2 overexpression in transgenic plants could explain this remodelling through its effects on PC synthesis.

## Introduction

*De novo* fatty acid synthesis in plants takes place in the plastid, with acetyl-coenzyme A (acetyl-CoA) the main precursor. This metabolite is primarily generated from pyruvate through the action of the plastidial pyruvate dehydrogenase complex (PDH), which catalyses the oxidative decarboxylation of pyruvate to produce acetyl-CoA, CO_2_ and NADH^1^. The PDH complex contains 3 subunits: E1 or pyruvate dehydrogenase, E2 or dihydrolipoyl acetyltransferase, and E3 or dihydrolipoyl transhydrogenase. Of these, E2 requires a lipoic acid (LA; 6,8-dithiooctanoic acid or 1,2-dithiolane-3-pentanoic acid) prosthetic group to be functional, a sulphur-containing enzyme co-factor that is essential for the catalytic activity of several key enzyme complexes involved in fundamental metabolic processes in most prokaryotic and eukaryotic organisms. Besides PDH, this prosthetic group is also required by α-ketoglutarate dehydrogenase, branched-chain α-ketoacid dehydrogenase, acetoin dehydrogenase, and the glycine cleavage complex (glycine decarboxylase)^2–5^.

LA is essentially a modified form of the short-chain fatty acid octanoic acid, carrying two thiol substituents at the Δ6 and Δ8 positions. In its oxidized form these thiols give place to a disulphide bond forming a 1,2-dithiolane ring. This co-factor is synthesised by two consecutive reactions: in the first step, an octanoyltransferase (LIP2) transfers the octanoyl moiety from octanoyl-acyl carrier protein (octanoyl-ACP) to the ε-amino group of a highly conserved Lys residue close to the N-terminus of apoproteins via an amide linkage, such as the aforementioned subunit E2^6^. Subsequently, the octanoyl moiety is transformed into LA by a lipoyl synthase (LIP1) by addition of two sulphur atoms to generate the lipoylated protein^7^.

The first sulphur atom is thought to proceed from S-adenosyl methionine (SAM). LIP1 is a member of the SAM enzyme superfamily that reduces SAM to generate 5-deoxyadenosyl radicals. LIP1 contains two distinct [4Fe–4S] clusters, one that is common to this enzyme family (RS cluster), while the second (auxiliary cluster) is exclusive of LIP1 that are thought to act as the direct sulphur donor^8–10^. Consequently, sulphur insertion takes place in two steps, in the first of which sulphur is obtained through SAM cleavage and inserted into C6 of an octanoyl chain through the action of the RS cluster. The auxiliary cluster then acts as the sulphur donor for the second insertion at C8. The hypothesis of the disassembly of the auxiliary cluster^8,9,11^ is not universally accepted, although some evidence does exist^12^. Therefore, LA is not synthesized and subsequently attached to the *apo*-forms of the enzymes but rather, its biosynthesis is performed *in situ* in its cognate proteins^13^.

LA biosynthesis has been extensively studied in prokaryotes and yeast (*Saccharomyces cerevisiae*), however little is known in plants. Two different pathways of lipoylation have been described in *Escherichia coli*, one involving *de novo* synthesis and other based on the scavenging of free lipoate^14^. As described above, *de novo* synthesis of LA occurs through the action of octanoyltransferase and lipoyl synthase (LIPB and LIPA in *E. coli*, respectively), a pathway relying on type II fatty acid synthase to produce a C8 intermediate in order to generate octanoyl-ACP^15^. Nevertheless, an alternative lipoylation pathway has been identified in *E. coli* whereby free lipoate or octanoate from the media is attached through the action of a lipoate protein ligase (LPLA)^7,17^. This mechanism requires the activation of lipoate to the lipoyl-AMP intermediate before it can be bound to the cognate proteins^16^.

Despite the importance of the lipoyl prosthetic group in many central metabolic enzymes, little is known about its synthetic pathway in plants. In this regard, PDH is the only LA-dependant enzyme whose location is not restricted to the mitochondria but also found in plastids^18,19^. In mitochondria, lipoylation occurs via LIP2 and LIP1^20,21^, although two mitochondrial LPLAs were found in *Arabidopsis thaliana* (*At*LPLA)^22^ and *Oryza sativa* (*Os*LPLA)^23^. These enzymes are also probably inducing lipoylation in this organelle, although their physiological relevance is not fully understood. In *A. thaliana* plastids, two redundant octanoyltransferases (*At*LIP2p and *At*LIP2p2) and an essential lipoyl synthase (*At*LIP1p) have been identified^24^. However, there is no evidence of a plastidial LPLA^24^, although LA biosynthesis is thought to use the octanoyl-ACP from fatty acid biosynthesis as a unique substrate. Hence, the synthesis of LA in plastids relies on the synthesis of a C8 fatty acid, which requires PDH activity to provide a carbon supply. Accordingly, LA biosynthesis is likely to interfere in fatty acid biosynthesis. This point has been recently confirmed through characterising Arabidopsis plastidial octanoyltransferase mutants by lipidomic analysis^25^. In the present study, two plastidial lipoyl synthase genes from sunflower were initially identified (*HaLIP1p1* and *HaLIP1p2*), overexpressed in a heterologous host (*E. coli*), their tertiary structure modelled by molecular docking, and their expression levels measured. Thereupon, after confirming the *in vivo* activity of these enzymes by their successful complementation of bacterial mutants lacking LIPA, fatty acids and lipids from transgenic Arabidopsis seeds overexpressing *Ha*LIP1p1 or *Ha*LIP1p2 were characterized by gas chromatography (GC-FID) and ultra-high performance liquid chromatography (HPLC) coupled to quadrupole-time of flight mass spectrometry (LC-HRMS2).

## Materials and Methods

### Cloning the cDNAs encoding HaLIP1p1 and HaLIP1p2

The plastidial lipoyl synthase (LIP1p) protein sequence from *A. thaliana* (At5g08415) was used to identify mRNAs encoding sunflower LIP1p homologues in a public sunflower genome database (Sunflower genome portal, Heliagene - https://www.heliagene.org/)^26^. Two cDNAs encoding two putative plastidial lipoyl synthases were identified (named *HaLIP1p1* and *HaLIP1p2*). Polymerase chain reaction (PCR) fragments encoding the entire putative sunflower lipoyl synthases were amplified using specific primers: *Ha*LIP1p1-*Bam*HI-F/*Ha*LIP1p1-*Hind*III-R and *Ha*LIP1p2-*Sph*I-F/*Ha*LIP1p2-*Hind*III-R (Supplementary Table S1; all the primers were synthesized by Eurofins MWG Operon, Germany). These fragments were then cloned into the pMBL-T vector (Canvax, Córdoba, Spain) and the nucleotide sequences were confirmed by sequencing (SECUGEN, Madrid, Spain). The identity of the clones was confirmed using the BLAST algorithm^27^ and full-length sequences were obtained by including the ATG and STOP codons. The sequences were deposited in GenBank under the accession numbers KU882090 (*HaLIP1p1*) and KU882091 (*HaLIP1p2*).

### Protein sequence analysis

Homologous sequences retrieved from NCBI (www.ncbi.nlm.nih.gov) using the BLASTp program were aligned with the predicted *Ha*LIP1p1 and *Ha*LIP1p2 proteins using the default settings of the ClustalX v.2.0.10 program^28^. This alignment was used to produce a phylogenetic tree with the MEGA6 software^29^ and a bootstrap analysis with 1000 replicates was used to support the tree nodes^30^. Signal peptides were identified using the network-based programs TargetP V1.1^31^, iPSORT^32^ and Predotar^33^, which localized both proteins to the chloroplast and detected the N-terminal plastidial targeting peptides. To study the conserved residues throughout evolution, alignments were made with different phylogenetic groups (Dicots: *Arabidopsis thaliana*-Brassicaceae*, Ricinus communis*-Euphorbiaceae*;* Monocots: *Oryza sativa*- Poaceae; and Clubmosses: *Selaginella moellendorffii*-Selaginellaceae;) using the ClustalX v.2.0.10 and BioEdit programs. To detect residues potentially involved in the catalytic activity of *Ha*LIP1p1 and *Ha*LIP1p2, their sequences were aligned with known lipoyl synthases from *Mycobacterium tuberculosis* (*Mt*LIPA)^12^ and *Thermosynechococcus elongatus*^10^, the structures of which have been crystallized.

### Modelling the three-dimensional structure of HaLIP1p and molecular docking

Homology modelling studies were performed using the Swiss Model server^34^ (http://swissmodel.expasy.org/) and the tertiary structure was modelled using the *Mt*LIPA X-ray structure as a reference^12^. As both *Ha*LIP1p1 and *Ha*LIP1p2 basically displayed the same structure, the *Ha*LIP1p1 model was used for the molecular docking prediction. Molecular docking was performed using the SwissDock server^35,36^ with lipoyl-Lys (ZINC12494640) and 5′-deoxyadenosine radical (ZINC01999286) as substrates. The docking model was visualized and analysed using the UCSF Chimera program^37^.

### Real-time quantitative PCR

Sunflower cDNAs from developing seeds (12, 14, 18, 20, 25, and 28 days after flowering – DAF) and other vegetative tissues (roots, stems, cotyledons, and leaves) were used to determine the levels of *HaLIP1p1* and *HaLIP1p2* gene expression by real time quantitative PCR (RT-qPCR). The reactions were performed in a CFX96^TM^ Real-Time PCR Detection System (Bio-Rad) using SYBR Green I (QuantiTect SYBR Green PCR Kit, Quiagen, Crawley, UK) and a specific primer pair designed for each gene: *Ha*LIP1p1qpcr-F/*Ha*LIP1p1qpcr-R; and *Ha*LIP1p2qpcr-F/*Ha*LIP1p2qpcr-R (Supplementary Table S1). The reaction mixture was heated to 94 °C for 10 min and then subjected to 50 PCR cycles of 94 °C for 30 seconds and 57 °C for 30 seconds, with a final extension period at 72 °C for 1 minute. Efficiency curves were drawn up using sequential of the cDNAs and the Livak method^38^ was applied to calculate the relative expression of the samples. The *HaActin*-qpcr-F4 and *HaActin*-qpcr-R4 primers (Supplementary Table S1) were used to amplify the sunflower *HaAct1* actin gene (GenBank Accession FJ487620) as a reference to normalize the expression values.

### Expression and purification of the recombinant proteins in *E. coli*

*HaLIP1p1* and *HaLIP1p2* were cloned into the pQE-80L expression vector (Qiagen, Hilden, Germany) that contains an N-terminal (His)6-tag to facilitate protein purification. PCR primers were designed to clone the genes encoding the mature proteins without a signal peptide: *Ha*LIP1p1-B-*Bam*HI-F/*Ha*LIP1p1-*Hind*III-R for *Ha*LIP1p1; and *Ha*LIP1p2-B-*Bam*HI-F/*Ha*LIP1p2-*Hind*III-R for *Ha*LIP1p2 (Supplementary Table S1). Accordingly, *Bam*HI and *Hind*III digested PCR products were cloned into the *Bam*HI:*Hind*III sites of pQE-80L and the constructs (pQE-80L::*Ha*LIP1p1 and pQE-80L::*Ha*LIP1p2) were confirmed by sequencing. Cultures of *E. coli* XL1-Blue strain harbouring the recombinant plasmids were grown at 37 °C in LB media (1% Bacto Tryptone, 0.5% yeast extract, 1% NaCl, pH 7) supplemented with 50 µg/mL ampicillin and expression of the recombinant protein was induced by isopropyl β-D-1-thiogalactopyranoside (IPTG, at a final concentration of 0.5 mM) when the culture reached an OD_600nm_ value of 0.4. After 4 hours the cells were harvested by centrifugation (4,000 rpm for 20 min) and then resuspended in 16 mL binding buffer (20 mM sodium phosphate [pH 7.4], 500 mM NaCl, 20 mM imidazole). The cells were disrupted by sonication (70° amplitude 10 s pulses with 10 s cooling on ice, 20 cycles in total) and a cleared lysate was obtained by centrifugation of the disrupted cell solution at 13,000 rpm and at 4 °C for 10 min. The soluble fraction was used to purify the recombinant proteins using the His SpinTrap Kit (GE Healthcare, UK) according to the manufacturer’s protocol.

### Gel electrophoresis of His-tagged recombinant proteins and western blots

Aliquots of the different eluted fractions (15 µL) were mixed with SDS-PAGE loading Buffer 2X(Tris-HCl 62.5 mM, DTT 50 mM, bromophenol blue 0.01%, glycerol 25%, SDS 2%) and 1 µL of β-mercaptoethanol, and then heated for 10 min at 90 °C. The proteins were separated by electrophoresis on 4–15% Mini-Protean TGX Gels (Bio-Rad) in SDS–PAGE Running Buffer (25 mM Tris-Base, 192 mM glycine, 0.1% SDS) and the gels were stained with 0.1% Coomassie R-250 in 40% ethanol and 10% acetic acid, visualizing the proteins after several washes. For western blotting, the proteins were transferred to a polyvinylidene difluoride (PVDF) membranes (TransBlot TurboTM Mini PVDF Transfer Packs Bio-Rad) in a TransBlot TurboTM Transfer Starter System (Bio-Rad) and the membranes were blocked for 1 hour with a solution of milk 5% (w/v) in Tris-buffered saline solution (TBS) with mild shaking. The membrane was then washed three times with TBS and incubated at 4 °C for 2 hours with anti-polyHistidine antibodies (1/2000 dilution: Sigma-Aldrich). The (His)_6_-tagged recombinant proteins were then visualized using the ECL Western Blotting Detection Kit (Amersham Bioscience).

### Fatty acid analysis in *E. coli*

The fatty acid composition of transformed *E. coli* cells expressing the recombinant mature *Ha*LIP1p1 and *Ha*LIP1p2 proteins was analysed in 25 mL cultures of *E. coli* XL1-Blue cells harbouring different constructs (empty pQE-80L expression vector, pQE-80L::*Ha*LIP1p1 and pQE-80L::*Ha*LIP1p2), both induced and non-induced, grown at 37 °C with shaking for two hours. Then cells were harvested by centrifugation (4000 rpm for 20 min) and the pellets washed twice with distilled water (2 mL). A methylation mixture (3 mL of ~1.25 M HCl in methanol: Fluka, Germany) was added to each sample and heptadecanoic acid (17:0, 150 µg) was added as an internal standard, allowing methylation to take place for 1 hour at 80 °C. Subsequently, 1 mL heptane was added to extract the methyl esters, the upper phase was washed with 2 mL Na_2_SO_4_ (6.7%) in new tubes and the resulting solvent was evaporated with nitrogen gas. Finally, methyl esters were resuspended in 200 µL heptane and gas chromatography (GC) analysis was performed as described previously^39^.

### Functional complementation of an *E. coli* ΔlipA strain

To study the genetic complementation of *Ha*LIP1p1 or *Ha*LIP1p2, a kanamycin resistant *E. coli lipA* strain (JW0623) with a defective LA synthase was obtained from the Coli Genetic Stock Center (CGSC). Transformants with the different plasmids (empty pQE-80L expression vector, pQE-80L::*Ha*LIP1p1 and pQE-80L::*Ha*LIP1p2) were generated by electroporation and the cells were incubated at 37 °C overnight on LB-agar plates supplemented with 50 μg/mL ampicillin for plasmid selection and 30 μg/mL kanamycin for mutant selection. The resulting colonies were inoculated in 30 mL M9 glucose minimal medium and grown at 37 °C overnight. These bacteria were then inoculated at 0.1 OD_600nm_ in 5 mL tubes with M9 supplemented with ampicillin, kanamycin and 0.5 mM IPTG for plasmid induction. The mutant *E. coli* transformed with pQE-80L was used as a negative control, with and without LA supplementation (50 ng/mL). The OD was measured every 90 minutes over the first 9 hours, and after 24 and 30 hours, and the mean of three independent experiments was used to calculate each OD value.

### Generation of transgenic plants and vector constructions

The entire open reading frame of the sunflower plastidial lipoyl synthases was cloned into the binary pBIN19-35S vector, which contains the 35S promoter from cauliflower mosaic virus (CaMV). The construct was cloned into the restriction sites created by PCR with specific pairs of primers: *Ha*LIP1p1-*Bam*HI-F/*Ha*LIP1p1-*Hind*III-R and *Ha*LIP1p2-*Xba*I-F/*Ha*LIP1p2-*Hind*III-R (Supplementary Table S1). The CAMV-35S-F and pBIN19-R vector primers were used for PCR screening (Supplementary Table S1) and the constructs were sequenced to confirm their identity. These constructs were then used to transform the CaCl_2_ competent *Agrobacterium tumefaciens* strain GV 3101.

*A. thaliana* Columbia (Col-0) ecotype plants were grown in a growth chamber under controlled growth conditions: 22 °C day/20 °C night, 60% humidity, and a 16 h, 250 micromol m^−2^ s^−1^ photoperiod. Transgenic Arabidopsis lines were generated by floral dip transformation, as described previously^40^. Seeds from transformed plants were selected by germination in MS medium supplemented with kanamycin (50 µg/mL), as described previously^41^. Insertion of the Sunflower gene into the Arabidopsis genome was screened by PCR, and the expression of both the *Ha*LIP1p1 and *Ha*LIP1p2 constructs in transgenic plants was confirmed by RNA extraction, cDNA synthesis and PCR. Third generation (F3) seeds of confirmed transgenic Arabidopsis were used to analyse the lipid composition.

### Fatty acid analysis of transgenic Arabidopsis seeds

Three replicates of 10 mg of mature seeds from wild-type and F2 transgenic plants overexpressing *Ha*LIP1p1 and *Ha*LIP1p2 were used for total lipid extraction. Lipids from the seeds were extracted using glass beads in a Precellys homogenizer (Precellys 24, Ozyme) 3 cycles for 30 s at 6000 rpm, and then 1 mL hexane:isopropanol (2:1) was added. After solvent evaporation, the lipids were resuspended in chloroform:methanol (1:1) and methylated with 5 µL of TMAH solution (Sigma-Aldrich), using Decane (50 µL) to stop the reaction. Finally, 20 µL of the upper phase was used to analyse the fatty acid methyl esters (FAMEs).

### Lipidomic analysis of transgenic seeds

Lipids were extracted as described elsewhere^42^ with some modifications. Samples of ice-dried *Arabidopsis seed* (20 mg) were transferred to a 2 mL screw cap tube and the lipids were extracted with 1 mL of a chloroform/methanol (2:1) mix (1 mL) containing 1 mM BHT and 400 µL H_2_O. After a 2 hour incubation on ice, the samples were centrifuged at 13000 rpm for 5 min at 4 °C, the lower phase was transferred to new tubes and the extraction was repeated. The lower phases were dried under nitrogen and the lipids extracted were solubilized in 200 µL isopropanol. A 1:4 dilution was used to perform lipidomics based on ultra-high performance liquid chromatography (HPLC) coupled to quadrupole-time of flight mass spectrometry (LC-HRMS2), as described previously with minor variations^43^. LC was performed on an HPLC 1290 (Agilent Technologies) and 1 µL of the lipid samples was separated at 55 °C on a C18 Hypersil Gold column (100 × 2.1 mm, 1.9 μm: Thermofisher) using an elution gradient consisting of a solution of 10 mM of ammonium formate and 0.1% formic acid (ACN:H_2_O, 60:40, v/v; solvent A), and a solution of 10 mM of ammonium formate and 0.1% formic acid (IPA:ACN:H_2_0, 90:8:2, v/v; solvent B). LC-electrospray ionization (ESI)-HRMS^2^ analysis were achieved by coupling the LC system to an Agilent 6538 hybrid quadrupole time-of-flight (QToF) high definition mass spectrometer (Agilent Technologies) equipped with a dual source ESI. MassHunter B.07 software was used to control the data acquisition parameters and the mass spectra were acquired using dual ESI in positive and negative ion mode.

The chromatogram builder was used applying a minimum time span of 0.10 min, a minimum height of 1.0 × 10^3^ and a m/z tolerance of 5 ppm. Chromatogram deconvolution was achieved using the local minimum search algorithm. The chromatographic threshold was 30.0%, the search minimum in the retention time (rt) range of 0.05 min with a minimum relative height of 5% and a minimum ratio of peak top/edge of 2. The peak duration range was from 0.05 to 3 min. MS2 scans were paired using an m/z tolerance range of 0.05 Da and a rt tolerance range of 0.1 min. Isotopologues were grouped using the isotopic peak grouping algorithm with a m/z tolerance of 0.008 and rt tolerance of 0.3 min. A peak alignment step was performed using the join aligner module: m/z tolerance = 0.008, weight for m/z = 50, weight for rt = 50, absolute rt tolerance 2 min. The peak finder module was used with an intensity tolerance of 10%, a m/z tolerance of 0.008 and a rt tolerance of 1.0 min. The resulting peak list was filtered using the Peak list rows filter module with a minimum of 2 peaks in a row, a minimum peaks in an isotope pattern of 2 and by keeping only peaks with MS/MS spectra using MS2 peak filer. The peak list was annotated using a combination of two databases, LipidMatch^44^ and LipidBlast^45^. The data were also subjected to a principal component analysis to explore the differences in lipid profiles among the seeds overexpression both sunflower seeds and the wild-type seeds.

A statistical analysis was performed using Metaboanalyst v4.0, and a Pearson-correlation analysis or an unsupervised principal component analysis (PCA) was carried out to determine the statistical significance (Supplementary Data). Heat maps were drawn up to test any significant differences between samples (p < 0.05).

### Statistical analysis

Origin Pro 8 software (OriginLab Corporation, Northampton, USA) was used for the statistical analysis. The number and nature of replicates for each figure and table are indicated in their corresponding legends. An analysis of variance (ANOVA) and a Tukey’s test were used to determine the significant differences of the means at a probability level of 5% (p < 0.05).

## Results

### Cloning and sequence analysis of two sunflower lipoyl synthases

Two coding sequences were identified in the Heliagene database based on their homology to the Arabidopsis LIP1p protein (At5g08415). A 1077 bp (*HaLIP1p1*) and 1149 bp (*HaLIP1p2*) PCR product were amplified from 28 DAF developing sunflower seed mRNA, and both were sequenced and found to have essentially the same nucleotide sequences as those from the genome sequencing project (*HaLIP1p1*, HanXRQChr05g0142151; *HaLIP1p2*, HanXRQChr12g0358451). The minor differences detected in these sequences were the lack of three nucleotides in a non-catalytic region of *Ha*LIP1p1 and changes in several nucleotides in the N-terminal signal peptide of *Ha*LIP1p2. The protein sequences encoded by the cDNAs of both genes were deduced using bioinformatics tools, containing 359 (*Ha*LIP1p1) and 382 (*Ha*LIP1p2) residues. Chloroplast transit peptides of 61 (Lys61) and 84 (Lys84) residues at their N-terminus were identified in *Ha*LIP1p1 and *Ha*LIP1p2 respectively, (Supplementary Fig. S1), with both transit peptides rich in Asn, Pro, Ser, and Thr. After proteolysis at the marked cleavage site, the mature proteins were both predicted to contain 229 residues, with a theoretical molecular mass of 33.1 kDa, and with a theoretical pI of 7.69 in the case of *Ha*LIP1p1 and 6.19 for *Ha*LIP1p2.

**Figure 1:**
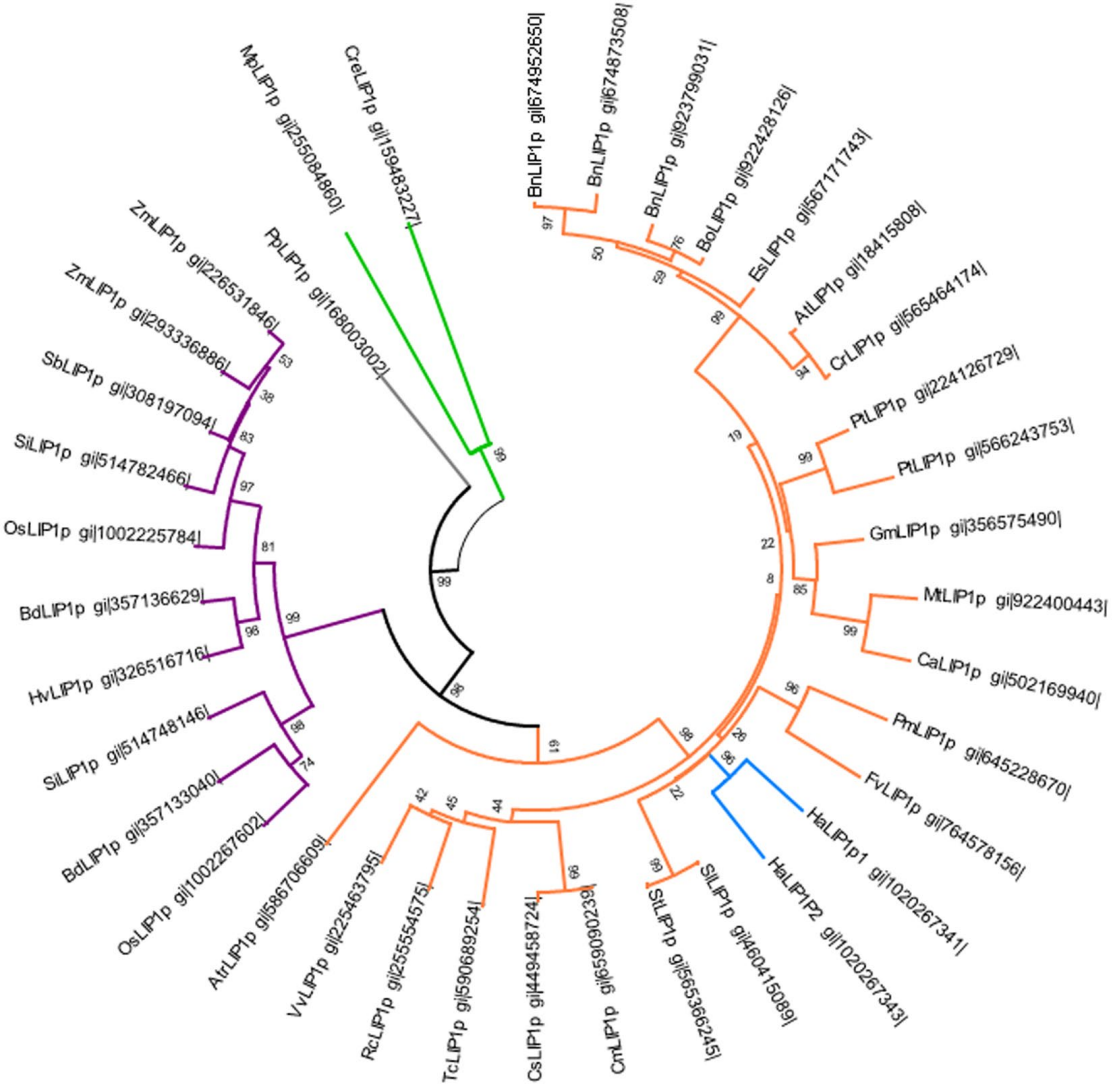
Phylogenetic tree of plant plastidial lipoyl synthase enzymes. The plant species included in the phylogenetic tree are: At, *Arabidopsis thaliana*; Atr, *Amborella trichopoda*; Bd, *Brachypodium distachyon*; Bn, *Brassica napus*; Bo, *Brassica olerac*ea; Ca, *Cicer arietinum*; Cc, *Cynara cardunculus*; Cm, *Cucumis melo*; Cr, *Capsella rubella*; Cs, *Cucumis sativus*; Es, *Eutrema salsugineum*; Fv, *Fragaria vesca*; Gm, *Glycine max*; Hv, *Hordeum vulgare*; Mp, *Medicago truncatula*; Os, *Oryza sativa*; Pm, *Prunus mume*; Pp, *Physcomitrella patens*; Pt, *Populus trichocarpa*; Rc, *Ricinus communis*; Sb, *Sorghum bicolor*; Si*, Setaria italica*; Sl, *Solanum lycopersicum*; St, *Solanum tuberosum*; Tc, *Theobroma cacao*; Vv, *Vitis vinifera* and Zm, *Zea mays*. The Green algae Cre, *Chlamydomonas reinhardtii* and Mp, *Micromonas pusilla* were used as an outgroup to root the tree (in green). The purple subtree represents monocots, while *Ha*LIP1p1 and *Ha*LIP1p2 (blue) are included in the dicots group (orange). The grey subtree includes the Bryophyta species.

A phylogenetic tree was generated for the novel sunflower Lip1p genes based on the predicted amino acid sequences and those from other known homologous plant proteins (Fig. 1). The dendrogram shows both proteins in a separate group, the Asteraceae family, and very close to those from the Solanaceae family, which includes *Solanum lycopersicum* and *S. tuberosum* (tomato and potato respectively), and those from the Rosaceae that include *Prunus mume* (the Chinese plum) and *Fragaria vesca* (wild strawberry).

An alignment of the two deduced amino acid sequences with those from different phylogenetic groups (*A. thaliana*, *Ricinus communis*, *Oryza sativa* and *Sellaginella moellendorffii*) showed highly conserved domains (Supplementary Fig. S1). Since no plant lipoyl synthases have been crystalized, we based the crystal structure of a lipoyl synthase on that from the cyanobacteria *Thermosynechococcus elongatus* (*Te*LIPA)^10^ and on *Mt*LIPA *Mycobacterium tuberculosis*^12^, aligning the structures so that the predicted catalytic residues in *Ha*LIP1p1 and *Ha*LIP1p2 were evident and marked in the previous alignment. LIP1 enzymes contain two [4Fe-4S] clusters bound by three strictly conserved Cys residues, constituting the characteristic CX_3_CX_2_C motif of this family, the “RS cluster” and the “auxiliary cluster” with a CX_4_CX_5_C motif. Within the sunflower LIP1 enzymes, the “RS cluster” is formed by: Cys123, Cys127 and Cys130 in *Ha*LIP1p1; Cys146, Cys150 and Cys153 in *Ha*LIP1p2 (red box in Supplementary Fig. S1); and the “auxiliary cluster” by: Cys92, Cys97 and Cys103 in *Ha*LIP1p1; Cys115, Cys120 and Cys126 in *Ha*LIP1p2 (green box in Supplementary Fig. S1). “RS cluster” is also ligated to a highly unusual serine residue located close to the C-terminus, which corresponds to Ser338 in *Ha*LIP1p1 and Ser360 in *Ha*LIP1p2 (green arrow in Supplementary Fig. S1). This serine residue forms part of a conserved C-terminal R(S/T)S motif, which is followed by an aromatic residue in *Mt*LIPA, and that is also found in *Ha*LIP1p1 (R_337_SSY_340_) and *Ha*LIP1p2 (R_359_SSY_362_; in blue box in Supplementary Fig. S1).

Other residues characteristic of the RS protein superfamily were: (i) Asn223 and Glu225 in *Ha*LIP1p1 and Asn246 and Glu248 in *Ha*LIP1p2 (in orange boxes in Supplementary Fig. S1); (ii) a “GXIXGX_2_E” motif, corresponding to S_263_IMLGLGE_270_ (*Ha*LIP1p1) and S_286_IMLGLGE_293_ (*Ha*LIP1p2; orange box in Supplementary Fig. S1); and (iii) a conserved Met residue, Met265 in *Ha*LIP1p1 and Met288 in *Ha*LIP1p2 (as part of the “GXIXGX_2_E” motif, in Supplementary Fig. S1). Furthermore, Arg residues were also identified in the new sequences that could be involved in substrate recognition, Arg337 in *Ha*LIP1p1 and Arg359 in *Ha*LIP1p2 (orange arrow in Supplementary Fig. S1).

### Tertiary structure prediction and molecular docking

**Figure 2:**
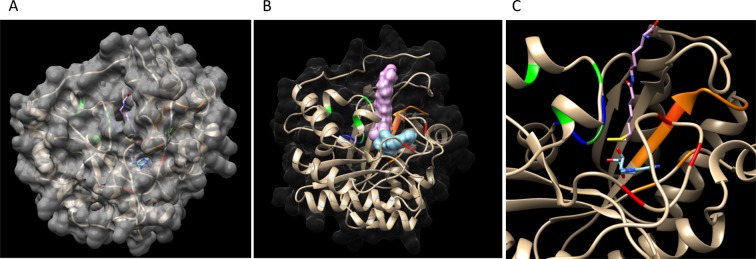
Model of the tertiary structure of *Ha*LIP1p. (**A**) Surface computing showing the gaps. (**B**) Ribbon diagram with both substrates in a 3D view, lipoyl-lys and 5′-deoxyadenosine interacting at the active centre. The hypothetical residues involved in the enzyme’s activity are in red (typical RS cluster involved in SAM reduction) and green (auxiliary cluster that acts as a donor of sulfur to form lipoic acid). Both substrates lipoyl-lys and 5′-deoxyadenosine are found in the gap formed between the two clusters. A Ser residue (green) forming part of R(S/T)Y motif (dark blue) is close to the auxiliary cluster, which enables the ligand to associate with the Cys of the cluster. The residues involved in SAM recognition are in orange. The peptide chain of lipoyl-lys remains outside the gap, with the sulfur atoms situated in the structure and 5′-deoxyadenosine staying close to the lipoyl group. (**C**) Wider angle of the active center of the protein with the substrates in a ribbon diagram.

Both lipoyl synthase tertiary structures from sunflower were modelled using the crystal X ray structure of *Mt*LIPA as a reference^12^. *Mt*LIPA shared 44.84% identity with *Ha*LIP1p1 and 46.45% with *Ha*LIP1p2, and both sunflower proteins shared 82.6% of identity such that the model generated was the same for both sequences. The predicted *Ha*LIP1p1 and *Ha*LIP1p2 structures consisted of a single domain with the overall folding producing a partial α8/β8 pattern TIM barrel (Fig. 2A). In the model, the auxiliary cluster is very close to the N-terminal region and the specific RS cluster is packed between the C- and N-terminal extensions. Both clusters, the auxiliary (three coordinated Cys in green in Fig. 2B,C) and RS cluster (three coordinated Cys in red in Fig. 2B,C) are spatially separated by 13 Å (in *Mt*LIPA), forming a gap in the structure where presumably the octanoyl chain from octanoyl-E2-PDH and the SAM interact. Furthermore, a docking model was constructed with the substrates lipoyl-lys and 5′-deoxyadenosine, substrates that were located in the gap formed between both clusters (Fig. 2B,C). The Ser residue (green in Fig. 2B,C) formed part of the R(S/T)Y motif (blue in Fig. 2B,C) and it lay close to the auxiliary cluster, enabling the ligand to access the Cys in the cluster. The residues involved in SAM recognition were located in the structure forming part of the gap where 5′-deoxyadenosine was positioned. The peptide chain of lipoyl-lys remained outside this gap, with sulphur atoms projecting into the structure and 5′-deoxyadenosine staying close the lipoyl group (Fig. 2B,C).

### Expression of lipoyl synthases in sunflower tissues

**Figure 3:**
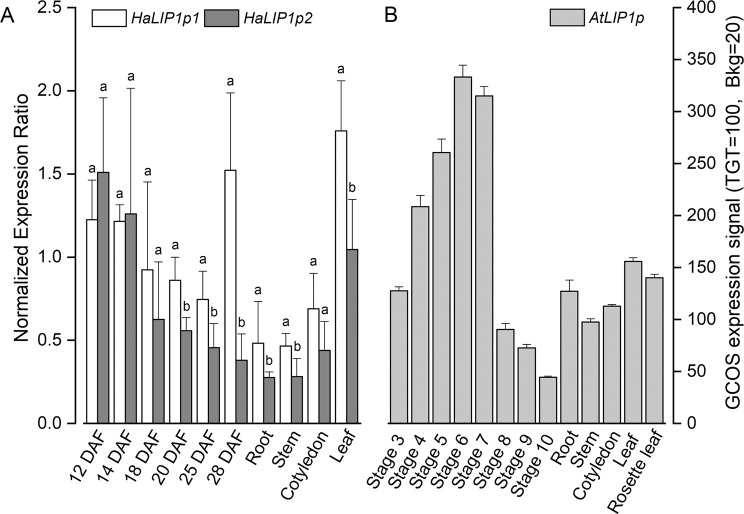
Expression of plastidial lipoyl synthases in *Helianthus annuus* and *Arabidopsis thaliana* tissues and seeds at different developmental stages. (**A**) The *HaLIP1p1* and *HaLIP1p2* genes expression normalized to *HaActin*. (**B**) The *Arabidopsis thaliana AtLIP1p* gene microarray data^46^. DAF, days after flowering; Stage 3, mid-globular to early heart embryos; Stage 4, early to late heart embryos; Stage 5, late heart to mid-torpedo embryos; Stage 6, mid to late torpedo embryos; Stage 7, late torpedo to early walking-stick embryos; Stage 8, walking-stick to early curled cotyledons embryos; Stage 9, curled cotyledons to early green cotyledon embryos. The data in panel A represent the mean values ± SD of three independent samples. ^(a, b)^At the 0.05 level, data are significantly different. The data in panel B represent the mean values ± SD of three independent samples.

The expression of *HaLIP1p1* and *HaLIP1p2* was analysed by RT-qPCR in different sunflower tissues, including developing seeds and vegetative tissues. The accumulation of transcripts from both genes follow a similar pattern in most tissues, so they are thought not be differentially regulated (Fig. 3A). A slight prevalence of the *HaLIP1p1* isoform, not statistically significant at early developing seeds (12–18 DAF) and seedling cotyledons, was detected in most of the tissues assayed (particularly in seeds at medium-late developmental stages, roots, stems and leaf tissue). When compared with *AtLIP1p* expression^46^ (Fig. 3B), both sunflower genes follow a similar pattern, taking in account that 12 DAF sunflower developing seeds are equivalent in oil accumulation to stage 6 Arabidopsis developing seeds.

### Complementation of JW0623 with the sunflower HaLIP1p1 and HaLIP1p2 genes

**Figure 4:**
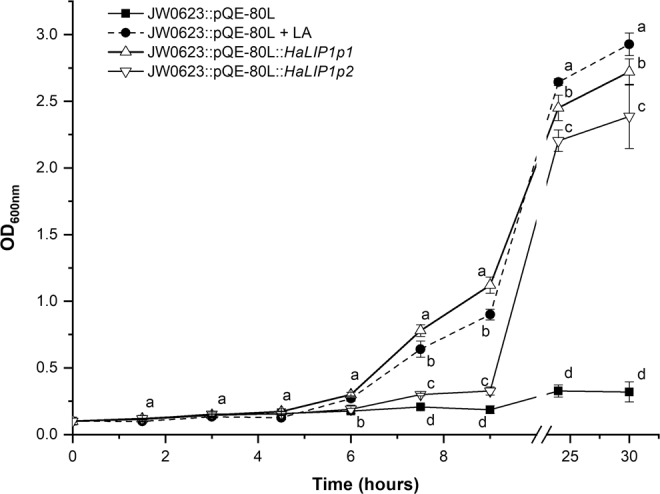
Complementation of LipA-deficient *E. coli* JW0623 in M9 minimal medium by transforming them with *Ha*LIP1p1 and *Ha*LIP1p2 in a pQE-80L expression vector, using *E. coli* cells transformed with the empty pQE-80L vector as a control: LA, lipoic acid supplementation (50 ng/ml). All cultures were induced with 0.5 mM IPTG and the break in the graph separates the growth during the first 9 hours, and the measurements at 24 and 30 hours. The data represent the means ± SD of three replicates and the different letters (a–d) reflect the mean statistical difference (p < 0.05) at each time point.

Mature *Ha*LIP1p1 and *Ha*LIP1p2, without signal peptide, could complement the *lipA* deficient *E. coli* JW0623 strain that has defective LA biosynthesis and that grows poorly in minimal medium. When this *E. coli* strain was transformed with *Ha*LIP1p1 its growth rate recovered completely, initially surpassing that of the *lipA* deficient cells fed with LA (Fig. 4). Conversely, the *Ha*LIP1p2 construct also complemented this auxotrophy in these experiments but with lower efficiency. In terms of the cell density in the stationary phase, the *E. coli* cells transformed with both LIP1p enzymes achieved similar densities to the cells that received a LA supplement and much higher than that of the *lipA* deficient mutant (Fig. 4). After 30 h of growth there were significant differences in the optical density of the cultures expressing sunflower LIP1p enzymes (Fig. 4), being in both cases significantly below that of the supplemented cells.

### Fatty acid analysis of *E. coli* expressing the HaLIP1p1 and HaLIP1p2 enzymes

The mature protein coding region of both sunflower lipoyl synthases was cloned into the pQE-80L inducible expression vector and the *E. coli* XL1-Blue strain was transformed with these vectors to achieve the heterologous expression of these enzymes. Mature *Ha*LIP1p1 and *Ha*LIP1p2 proteins were purified from the soluble fraction of these cell lysates using affinity columns, and both recombinant proteins had a molecular weight similar to that predicted (33.1 kDa; Supplementary Fig. S2). When the fatty acid composition of the cells expressing *Ha*LIP1p1, *Ha*LIP1p2 and the pQE-80L vector alone were analysed (Table 1), no significant changes in the total fatty acids per absorbance unit was obtained. However, qualitative modifications in terms of bacterial fatty acids composition were measured. Statistical significant differences were found after comparing the fatty acid profiles of the strain bearing the empty vector and the heterologous *Ha*LIP1p2 system, though the expression of *Ha*LIP1p1 did not produce significant changes. The expression of *Ha*LIP1p2 changed the bacterial fatty acids distribution indeed, decreasing the contents of 14:0 and 16:0 and increasing 18:1^Δ11^, therefore modifying the ratio of unsaturated to saturated fatty acids.

**Table 1:** Fatty acid composition (mol%) of *E. coli* cells containing the control and recombinant plasmids.

	pQE-80L	pQE-80L::*HaLIP1p1*	pQE-80L::*HaLIP1p2*
14:0	4.58 ± 0.92*	5.93 ± 0.13*	3.29 ± 0.04**
16:0	45.99 ± 0.31*	46.07 ± 0.14*	43.33 ± 0.48**
16:1^Δ9^	18.97 ± 1.54*	18.4 ± 0.35*	18.82 ± 0.12*
17:0Δ^a^	10.31 ± 1.45*	8.86 ± 0.20*	9.84 ± 0.20*
18:0	0.72 ± 0.10*	1.22 ± 0.15*	1.40 ± 0.70*
18:1^Δ11^	18.39 ± 1.21*	18.73 ± 0.17*	21.79 ± 0.21**
19:0Δ^a^	1.03 ± 0.14*	0.77 ± 0.12*	1.53 ± 0.53*
SFA^b^	51.30 ± 1.18*	53.23 ± 0.19*	48.02 ± 0.71**
UFA^c^	48.70 ± 1.83*	46.77 ± 0.19*	51.98 ± 0.71**
UFA/SFA	0.95 ± 0.04*	0.88 ± 0.06*	1.08 ± 0.03**
mg FAs/unit OD_600nm_	0.33 ± 0.02*	0.30 ± 0.02*	0.29 ± 0.02*

Fatty acids: 14:0, myristic acid; 16:0, palmitic acid; 16:1^Δ9^, palmitoleic acid; 17:0Δ, *cis*-9,10-methylenehexadecanoic acid; 18:0, stearic acid; 18:1^Δ11^, *cis*-vaccenic acid; 19:0Δ, *cis*-9,10-methyleneoctadecanoic acid. Cultures were induced with 0.5 mM IPTG and the data are the average ± SD of three independent samples (*Ha*LIP1p1 and *Ha*LIP1p2) and of six samples for the control cultures with pQE-80L. (*, **) At the 0.05 level, data are significantly different.

^a^C17 and 19 cyclopropanes derived from C16:1 and 18:1, respectively. ^b^Saturated fatty acids 14:0 + 16:0 + 18:0. ^c^Unsaturated fatty acids and their derivatives 16:1^Δ9^ + 17:0Δ + 18:1^Δ11^ + 19:0Δ.

### Fatty acid analysis of Arabidopsis seeds overexpressing the HaLIP1p1 and HaLIP1p2 enzymes

The total fatty acid composition of mature seeds from transgenic Arabidopsis overexpressing *Ha*LIP1p1 (*Ha*LIP1p1-ox) and *Ha*LIP1p2 (*Ha*LIP1p2-ox) was analyzed and compared to wild-type (Col-0) seeds. The overexpression of these enzymes failed to produce any significant changes in the fatty acid profile, although *Ha*LIP1p2 produced a slight increase, no statistically significant, in the 18:3^Δ9Δ12Δ15^ detected (Table 2). Hence, and in contrast to the described above modifications in transgenic bacteria, the unsaturated/saturated fatty acid ratio in both transgenic seed oils remained similar and unchanged when compared to wild-type seed oil.

**Table 2:** Fatty acids composition (mol%), thousand seeds weight (mg) and oil content (weight%) of wild type and overexpressing *Ha*LIP1p1 and *Ha*LIP1p2 *A. thaliana* plants.

	WT Col0	*Ha*LIPp1	*Ha*LIPp2
16:0	7.69 ± 0.05*	7.33 ± 0.06*	7.47 ± 0.07*
16:1^Δ9^	0.21 ± 0.04*	0.20 ± 0.03*	0.18 ± 0.05*
18:0	3.46 ± 0.11*	3.28 ± 0.14*	3.46 ± 0.02*
18:1^Δ9^	17.35 ± 0.07*	17.72 ± 0.05*	16.76 ± 0.22*
18:1^Δ11^	0.09 ± 0.03*	0.11 ± 0.01*	0.10 ± 0.02*
18:2^Δ9Δ12^	27.79 ± 0.25*	28.06 ± 0.18*	27.71 ± 0.04*
18:3^Δ9Δ12Δ15^	16.04 ± 0.04*	16.00 ± 0.07*	16.71 ± 0.20*
20:0	2.01 ± 0.05*	2.04 ± 0.01*	2.16 ± 0.03*
20:1^Δ11^	21.33 ± 0.18*	21.17 ± 0.08*	21.39 ± 0.03*
20:1^Δ13^	1.70 ± 0.01*	1.76 ± 0.01*	1.81 ± 0.03*
20:2^Δ9Δ12^	0.29 ± 0.02*	0.31 ± 0.02*	0.30 ± 0.01*
22:0	0.23 ± 0.01*	0.22 ± 0.01*	0.21 ± 0.05*
22:1^Δ13^	1.67 ± 0.05*	1.74 ± 0.02*	1.66 ± 0.03*
24:0	0.14 ± 0.08*	0.07 ± 0.03*	0.06 ± 0.01*
SFA^a^	13.53 ± 0.02*	12.93 ± 0.21*	13.38 ± 0.08*
UFA^b^	86.47 ± 0.02*	87.07 ± 0.21*	86.62 ± 0.08*
UFA/SFA	6.39 ± 0.01*	6.73 ± 0.13*	6.48 ± 0.05*
TSW (mg)	16 ± 1.4*	15 ± 1.3*	14 ± 1.3*
Oil (weight%)	35.0 ± 1.2*	34.9 ± 1.3*	35.0 ± 1.3*

Fatty acids: 16:0, palmitic acid; 16:1^Δ9^, palmitoleic acid; 18:0, stearic acid; 18:1^Δ9^, oleic acid; 18:1^Δ11^, asclepic acid; 18:2^Δ9Δ12^, linoleic acid; 18:2^Δ9Δ12Δ15^, linolenic acid; 20:0, arachidic acid; 20:1^Δ11^, gondoic acid; 20:1^Δ13^, paullinic acid; 20:2^Δ9Δ12^, eicosadienoic acid; 22:0, behenic acid; 22:1Δ13, erucic acid; 24:0, lignoceric acid. The data are the average ± SD of three independent samples. (*) At the 0.05 level, data are not significantly different.

^a^Saturated fatty acid: 16:0 + 18:0 + 20:0 + 22:0 + 24:0. ^b^Unsaturated fatty acids and their derivatives: 16:1^Δ9^ + 18:1^Δ9^ + 18:1^Δ11^ + 18:2^Δ9Δ12^ + 18:3^Δ9Δ12Δ15^ + 20:1^Δ11^ + 20:1^Δ13^ + 20:2^Δ9Δ12^ + 22:1^Δ13^. TSW: Thousand Seeds Weight.

### Lipidomics of transgenic seeds

**Figure 5:**
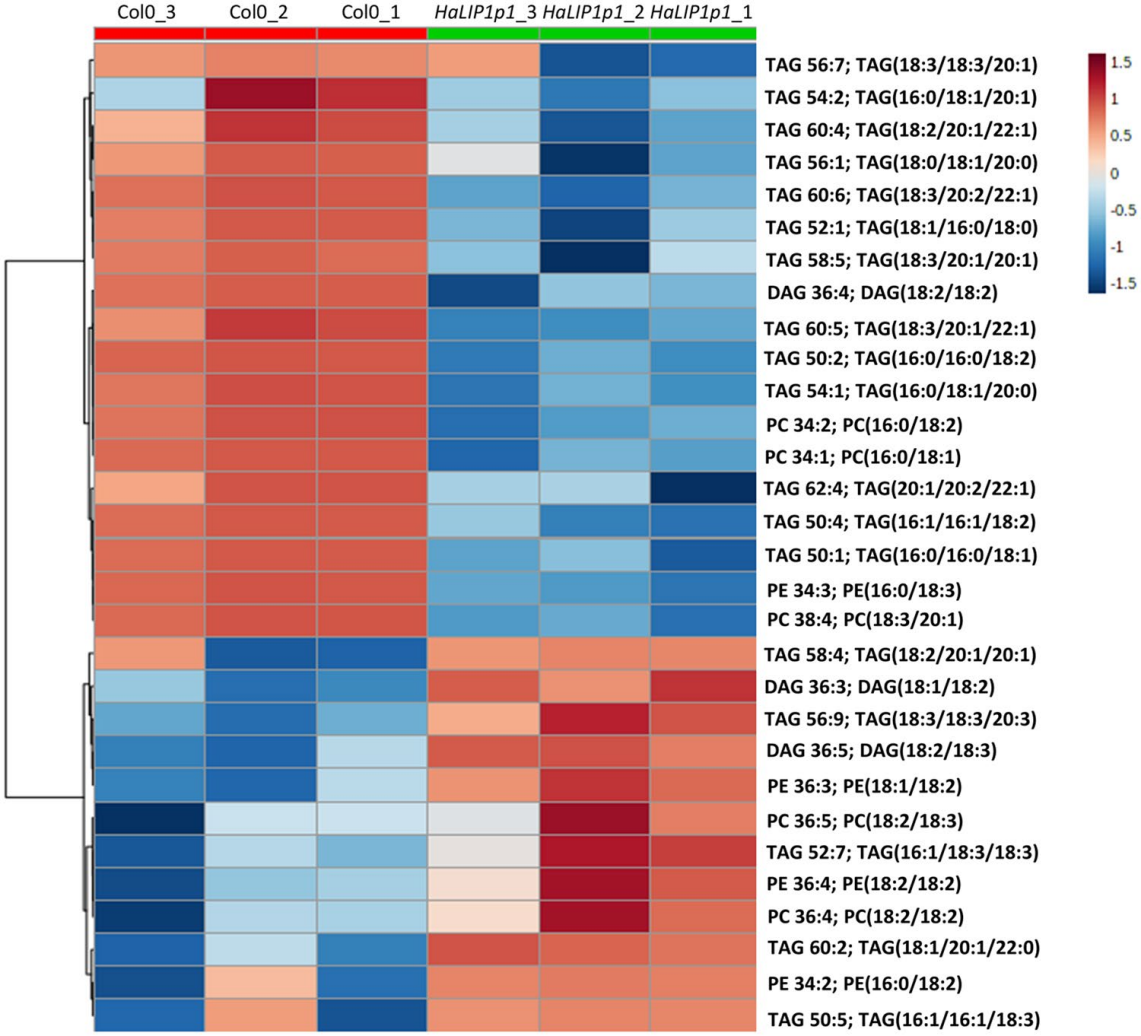
Heat map of the 25 lipid species showing more significant differences (right) comparing the WT Col-0 seeds (red) and transgenic seeds overexpressing *Ha*LIP1p1 (green). TOP 25 significant differences (p < 0.001) Anova and Tuckey non parametric. TAG, triacylglycerol; DAG, diacylglycerol; PC, phosphatidylcholine; and PE, phosphatidylethanolamine.

**Figure 6:**
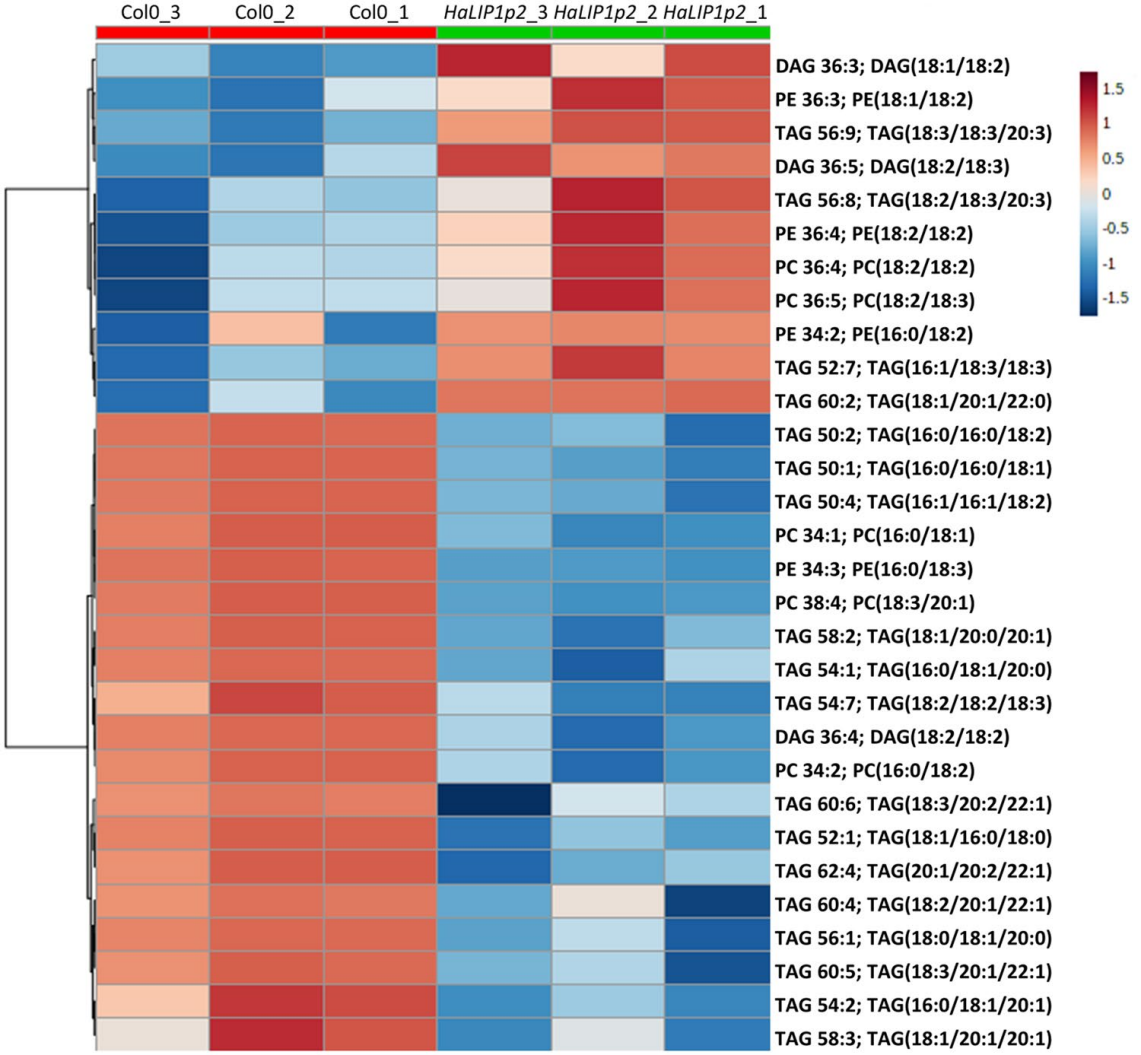
Heat map of the 25 lipid species showing more significant differences (right) comparing the WT Col-0 (red) and transgenic seeds overexpressing *Ha*LIP1p2 (green). TOP 25 significant differences (p < 0.001) Anova and Tuckey non parametric. TAG, triacylglycerol; DAG, diacylglycerol; PC, phosphatidylcholine; and PE, phosphatidylethanolamine.

**Figure 7:**
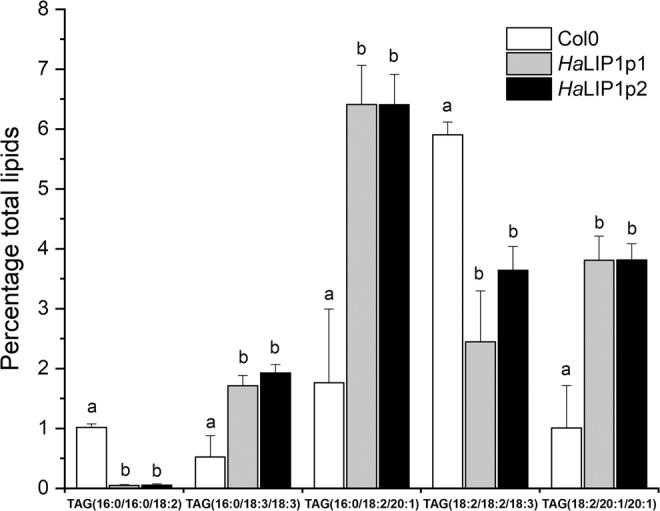
Representative TAG species of the differences found between control (Col0, in white) and transgenic (*Ha*LIP1p1, grey, and *Ha*LIP1p2, black) Arabidopsis mature seeds. Data are average values ± SD of three biological replicates. ^(a, b)^At the 0.05 level, data are significantly different.

**Figure 8:**
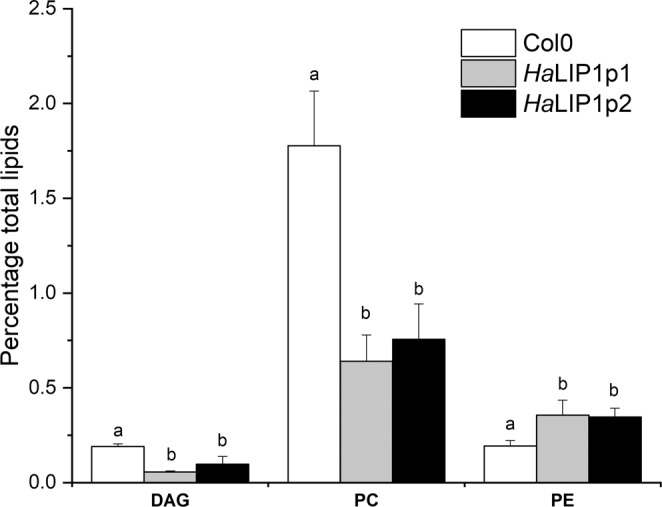
Diacylglycerol (DAG), phosphatidylcholine (PC) and phosphatidylethanolamine (PE) content (% total lipids) in control (Col0, in white) and transgenic (*Ha*LIP1p1, grey bars, and *Ha*LIP1p2, black bars) Arabidopsis mature seeds. Data are average values ± SD of three biological replicates. ^(a, b)^At the 0.05 level, data are significantly different.

Lipidomic analysis of control (Col-0) and transgenic Arabidopsis seeds expressing either *HaLIP1p1* or *HaLIP1p2* were performed, afterwards unsupervised principal component analysis (PCA) was employed in order to determine the experimental variation. The genotypes were separated according to their score plots, providing an overview of the differences among Col-0 and the transgenic plants regarding their seed oil composition (Supplementary Fig. S3). In both analysis, Col-0 versus *Ha*LIP1p1-ox and Col-0 versus *HaLIP1p2-ox*, clear separation between overexpression lines and the control highlighted their differences in terms of lipid composition. The heat maps of the statistically significant variations in lipids species were obtained, comparing the effects of *Ha*LIP1p1 and *Ha*LIP1p2 overexpression with the wild-type seeds. In Figs. 5 and 6 the heat maps showing the 25 species displaying the most significant differences were shown. In both cases, there was a clear change in the relative content of some triacylglycerol (TAG), PC, PE and DAG species relative to the wild-type. The species showing the most relevant statistical differences in the two transgenic lines were extracted in Figs. 7 and 8. With regard to TAG species, overexpression lines shown higher content of 16:0/18:3/18:3, 16:0/18:2/20:1, and 18:2/20:1/20:1 TAG, whereas they displayed lower 16:0/16:0/18:2 and 18:2/18:2/18:3 TAG comparing to control line (Fig. 7). Similarly, differences have been identified in other important lipids, seed oil from *Ha*LIP1p1-ox and *Ha*LIP1p2-ox Arabidopsis plants presented lower percentage of PC and DAG together with total increase in PE (Fig. 8).

## Discussion

Plastidial lipoyl synthase and octanoyltransferase are the enzymes involved in *de novo* LA biosynthesis within that organelle. LA is essential for the correct activity of the PDH complex, which catalyses the oxidative decarboxylation of pyruvate to produce acetyl-CoA, CO_2_ and NADH. Acetyl-CoA is the source of carbon for many synthetic pathways, including that responsible for the *de novo* synthesis of fatty acids. Therefore, better understanding this primary pathway could be important to provide a basic groundwork for further biotechnological approaches to modify lipid biosynthesis and accumulation in sunflower seeds.

### Sunflower lipoyl synthases

In the present work, two lipoyl synthase genes from sunflower were isolated and cloned. Both are nuclear genes encoding proteins with a N-terminal transit signal peptide, which is presumably removed upon transport into plastids or chloroplasts (Supplementary Fig. S1). Both signal sequences are rich in Asn, Pro and in particular, Ser and Thr residues, which are very common to such domains^47,48^. Indeed, these domains can be phosphorylated to bind to 14-3-3 proteins and to form a cytosolic complex for transport towards the chloroplast with HSP70 proteins^49^. Previous studies on sunflower proteins involved in fatty acid biosynthesis identified similar phosphorylation motifs, such as in FatA-type thioesterase^50^, the *Ha*FAD7 ω3-desaturase^51^, the condensing enzyme *Ha*KASIII^39^, and in the two β-hydroxyacyl-[ACP]-dehydratases *Ha*HAD1 and *Ha*HAD2^52^.

A phylogenetic tree was generated with the deduced amino acid sequences for *Ha*LIP1p1 and *Ha*LIP1p2, and with the chloroplast lipoyl synthases from different taxonomic groups, using two *Chlorophyta* species as out-groups to root the tree (Fig. 1). The location of the sunflower LIP1p proteins within the tree revealed a common origin for both proteins and a possible recent duplication. The dendrogram shows that the LIP1p proteins closest to the sunflower enzymes were those of Solanaceae, as expected, and Rosaceae.

The alignment of amino acid sequences of *Ha*LIP1p1 and *Ha*LIP1p2 with homologues from different phylogenetic groups identified highly conserved residues, indicating the strong conservation of lipoyl synthases during evolution (Supplementary Fig. S1). The overall secondary structure of both sunflower lipoyl synthases adopted a α8/β8 TIM barrel pattern (Fig. 2), which with certain variations relative to the common partial (α6/β6) TIM barrel typical of the RS superfamily^53^, and with N-terminal and C-terminal extensions^12^. Lipoyl synthase generates the lipoyl co-factor by inserting two sulphur atoms at C6 and C8 of an octanoyl chain bound to the E2 subunit of PDH. As mentioned before, the two [4Fe-4S] groups are directly involved in the reaction catalysed by this enzyme. LIP1 enzymes belongs to the radical SAM (RS) family of enzymes, which contains a [4Fe-4S] cluster bound by three strictly conserved Cys residues constituting the RS cluster characteristic of this family. Within the sunflower LIP1 enzymes, this cluster functions as a reductant in the cleavage of SAM to provide a sulphur atom and its insertion in the position Δ6 of octanoyl chain, generating 5′-deoxyadenosyl radicals. The second auxiliary cluster is specific to lipoyl synthases and it is thought to act as the direct sulphur donor for the second insertion at Δ8^8–10^. In accordance with their activity and our structural model, the arrangement of the RS and the auxiliary cluster is similar in the *Mt*LIPA and the sunflower LIP1 enzymes. The RS cluster of *Ha*LIP1p1 and *Ha*LIP1p2 is coordinated by a triad of Cys residues according to the CX_3_CX_2_C motif described for *Te*LipA^10^ and *Mt*LipA^12^. In the auxiliary cluster, three iron atoms are thought to be coordinated by three Cys residues in the CX_4_CX_5_C motif, while the fourth iron is coordinated by an unusual Ser residue^10,12^. This Ser is located near the C-terminal in the secondary structure, yet it stays close to the auxiliary cluster in our tertiary structure model. Thus, both clusters and the residues described above appear to form a structure that could nicely accommodate the substrate (octanoyl-Lys from octanoyl-E2-PDH) packed between SAM and the auxiliary cluster, the SAM lying adjacent to the RS cluster.

For molecular docking, lipoyl-lys (produced by insertion of the sulphur to form LA bound to Lys-E2-PDH) and 5′-deoxiadenosine (a radical produced after the loss of sulphur in SAM) were used as substrates (Fig. 2). Both substrates were positioned in the gap formed between the auxiliary and RS clusters. The mechanism proposed indicates that the active site is open, with the two clusters separated when no substrate is available. The octanoyl-E2-PDH substrate binds near to the N-terminal extension and the octanoyl chain is inserted into the gap between the two clusters. Subsequently, a conformational change takes place to close the two clusters, whereby the N-terminal extension and auxiliary cluster move towards the core of the partial TIM barrel. As a result, the distance between the clusters diminishes^12^. This change enables the cleavage of SAM and the first sulphur insertion at C6 of the octanoyl chain due to the action of the RS cluster. By contrast, the reductive cleavage of SAM generates methionine and a 5′-deoxyadenosyl radical. The function of the auxiliary cluster has not yet been fully elucidated but studies with ^34^S-labelled lipoyl synthase from *T. elongatus* suggest that both the sulphur atoms are transferred from a single lipoyl synthase molecule^8^, which may indicate the auxiliary cluster acts as the sulphur donor for the second insertion at C8. This hypothesis of the scarification of the auxiliary cluster is not universal accepted^8,9,11^, although there is some evidence to support this^12^.

A conserved Arg residue has been proposed to participate in the recognition of adenosyl ligands^54,55^, a phenomenon not previously observed in the RS superfamily. This Arg residue has been implicated in SAM recognition by *Te*LIPA^10^, and the equivalents in sunflower are Arg337 (*Ha*LIP1p1) and Arg359 (*Ha*LIP1p2). Furthermore, the unusual serine binding to the auxiliary [4Fe-4S] cluster (Ser338 in *Ha*LIP1p1 and Ser360 in *Ha*LIP1p2) may be involved in catalysis. Site-directed mutagenesis suggests this serine residue is essential for lipoyl group formation but not for the reductive cleavage of SAM^10^. An alignment of 590 lipoyl synthase sequences^56^ shows that the serine ligand is conserved in over 98% of these sequences, while the remaining sequences that lack this conserved serine appear to be eukaryotic splice variants.

The RT-qPCR data revealed similar expression patterns for the two sunflower plastidial lipoyl synthases genes (Fig. 3). *HaLIP1p1* is more strongly expressed in vegetative tissues and seeds from 20–28 DAF than *HaLIP1p2* and thus, neither gene appears to be temporally regulated during embryo development. These results suggest that both isoforms may be redundant and neither is tissue-specific. Indeed, both genes are expressed in all the tissues analysed, in contrast to the majority of genes involved in lipid biosynthesis and oil accumulation which are normally expressed more strongly in seeds, particularly around 18–19 DAF when lipid accumulation is at its highest in developing sunflower seeds^57^. This could be explained by the fact that *LIP1p* genes are essential enzymes for plant development. In Arabidopsis, the process of storage takes place during stages 5–6 of seed development, a period equivalent to 12 DAF in sunflower seeds, and both plants show similar lipoyl synthase expression during seed development and maturation.

### *E. coli* mutant complementation studies

To confirm the activity of both sunflower LIP1 enzymes *in vivo*, complementation of the in *E. coli* JW0623 strain was assessed, which carries a null *lipA* mutation in the LA synthase gene (Fig. 4). This mutant grows slowly in LB medium^58^ and its slow growth is further exacerbated in M9 minimal medium where no free LA is available for lipoate scavenging by LPLA. Only when LA is added to the medium can the *lipA* mutant recover a normal growth rate but when this mutant expressed either *Ha*LIP1p1 or *Ha*LIP1p2, its growth rate was also restored in M9 minimal medium without LA. Hence, both sunflower LIP1p enzymes are capable of achieving functional complementation. Despite this functional complementation, the heterologous expression of both sunflower lipoyl synthases in *E. coli* produced only minor, although statistically significant, changes in fatty acid composition, with only a slight increase in saturated fatty acids when *Ha*LIP1p2 was expressed. The expression of both enzymes reduces the growth rate of bacteria (data not shown), which could be due to altered LA metabolism by the bacteria, sequestering lipoate moieties in the E2 subunit of PDH and limiting their availability to other enzymes. There are several lipoylated proteins in *E. coli* other than PDH, such as α-ketoglutarate dehydrogenase (2OGDH), an enzyme essential for aerobic growth and a subunit of the glycine cleavage system of single carbon metabolism^59,60^. In bacteria, LA synthesis proceeds through a two-step reaction catalysed by the LIPA lipoyl synthase and the LIPB octanoyltransferase, both homologues of sunflower lipoyl synthase (*Ha*LIP1p1 and *Ha*LIP1p2) and octanoyltransferases. However, LIPB can be by-passed by a LPLA in *E. coli*, which can use free lipoate or octanoate in an ATP-dependent reaction to activate apo-subunits^6^. Therefore, excess PDH lipoylation without an increase in fatty acid synthesis could provoke a depletion of that co-factor, thereby impeding the synthesis of other enzymes and impairing bacterial growth.

In plastids, where no ligases have been identified, lipoylation of E2-PDH proceeds exclusively through the joint activity of LIP1p and LIP2p, both enzymes specific to plastidial E2-PDH. Arabidopsis has only one lipoyl synthase gene (*At*LIP1p) and its deletion is embryonic lethal, indicating that there is no alternative activity^24^. By contrast, two redundant octanoyltransferases have been identified that are simultaneously expressed in vegetative tissues and seeds, albeit in different ratios (*At*LIP2p1 dominates in leaves and roots and *At*LIP2p2 in siliques and flowers). Given the expression patterns observed here, we suggest that both lipoyl synthases are important not only for oil deposition in the seed but also, in vegetative tissues (Fig. 3), with some possible redundancy as seen for the two Arabidopsis octanoyltransferases. The strong identity between the two protein sequences (82.6% identity) would favour such redundancy.

### Overexpression of sunflower lipoyl synthases in transgenic Arabidopsis plants

Arabidopsis transgenic plants overexpressing *Ha*LIP1p1 or *Ha*LIP1p2 did not affect either plant development (Supplementary Fig. S4) or seed germination rates. Their seed fatty acid composition was not altered either (Table 2). Interestingly, lipidomic studies of transgenic seeds revealed a significant remodelling of TAG, diacylglycerides (DAG), phosphocholine (PC) and phosphoethanolamine (PE) species in Arabidopsis expressing the sunflower lipoyl synthase genes when compared with control plants. This was first show by the heat maps of variation of different species, in which were selected the 25 species displaying higher variations for *Ha*LIP1p1 and *Ha*LIP1p2 (Figs. 5 and 6). These figures clearly shown changes in lipid composition induced by the genes but it need further elaboration to reach general conclusions, so the most remarkable changes regarding TAG species are shown in Fig. 7 and those concerning other lipid species were summarized in Fig. 8. The expression of those genes altered the proportion of 7% of the TAG species, with important decreases of (16:0/16:0/18:2) and (18:2/18:2/18:3), specially the first one, which was barely detectable in the transgenic lines. The second one showed a decrease around 50% of the control value. The species increasing their proportion were (16:0/18:3/18:3), (16:0/18:2/20:1) and (18:2/20:1/20:1), which in all cases increased by 3 to 4-fold with respect to the control line (Fig. 7). The expression of both lipoyl synthases genes also induced variations on the content of DAG, PC and PE species. Thus, the content of DAG decreased by 45 to 70% and the PC 55 to 70%, with a concomitant increase of PE, which rose about 70–80% in both transformed plants (Fig. 8).

**Figure 9:**
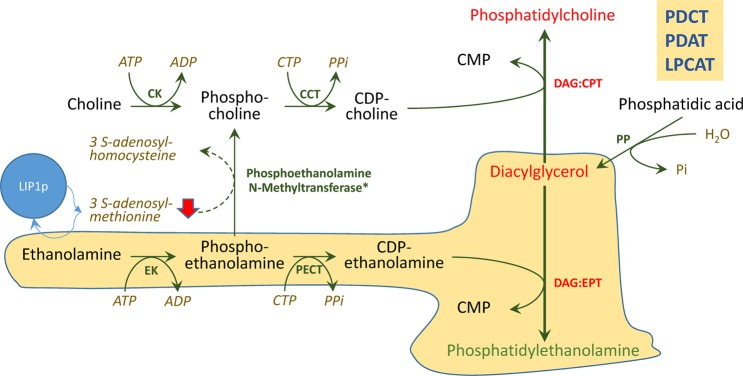
Scheme showing a possible explanation for the changes observed in the transgenic Arabidopsis plants overexpressing sunflower lipoyl synthases genes. CK, Choline kinase; CCT, Choline-phosphate cytidylyltransferase; DAG:CPT, Diacylglycerol cholinephosphotransferase; DAG:EPT, Diacylglycerol ethanolaminephosphotransferase; EK, Ethanolamine kinase; LPCAT, Lysophospholipid acyltransferase; PDAT, Phospholipid:diacylglycerol acyltransferase; PDCT, Phosphatidylcholine:diacylglycerol cholinephosphotransferase; PECT, Phosphoethanolamine cytidyltransferase; PP, Phosphatidate phosphatase.

The changes in lipid species composition induced by sunflower plastidial lipoyl synthase enzymes in Arabidopsis are not easy to interpret. Thus, the expected effect of the overexpression of those genes is the increment of the activity of the PDH complex due to a higher availability of LA cofactor. This increase would lead to higher availability of carbon and reduction equivalents for intraplastidial fatty acid synthesis and also a depletion of the substrates necessary for the synthesis of LA, such as the SAM. The higher availability of intraplastidial reducing equivalents would favour the desaturase reaction, which is dependent of it. This was observed in the E. coli expressing *Ha*LIP1p1(Table 1), but it was not evident when they were expressed in Arabidopsis, where there were not important changes in the desaturation ratios observed in transgenic plants with respect to control (Table 2). The modifications detected in glycerolipid species mostly involved changes in the proportion of neutral and polar lipid species (DAG, PC and PE) and different distribution of oleic acid derivatives in TAG species. In this regard, later changes could be caused by the reduction of PC and DAG, which species closely related with TAG synthesis^1^ and could provoke modifications in the contribution of the different enzymes involved in the channelling of fatty acids from PC and acyl-CoA pools to TAG, like PDCT, PDAT or LPCAT to the distribution of fatty acids in TAG species during their assembly. It should be noted that the glycerolipid species altered in the transgenic plants (DAG, PC and PE) are related. DAG is a common precursor in the biosynthesis of both PC and PE by the amino alcohol phosphotransferase enzyme (AAPT). AAPT catalyse PC biosynthesis from CDP-choline as a substrate (Diacylglycerol Cholinephosphotransferase, DAG:CPT) and PE, through CDP-ethanolamine activity (Diacylglycerol Ethanolaminephosphotransferase, DAG:EPT) (Fig. 9). Both CDP derivatives originate from phosphocholine or phosphoethanolamine due to choline-phosphate cytidyltransferase (CCT) or CTP:phosphoethanolamine cytidyltransferase (PECT), respectively. Even more, this phosphoethanolamine is converted to phosphocholine by the phosphoethanolamine N-methyltransferase, which consumes three SAM molecules, the co-factor also used by lipoyl synthases. In this context, as the physiological requirement for LA is extremely low, an excess of lipoyl synthase activity could provoke a temporal depletion of SAM when sunflower lipoyl synthases are overexpressed within the plastid. This essential co-factor is not produced in this organelle but in the cytosol using the methionine biosynthesized in plastids. Indeed, the influx of SAM into plastids could therefore reduce the pool of cytoplasmic SAM, dampening the synthesis of PC from PE (Fig. 9). This would provoke a reduction in PC with a concomitant effect on fatty acids assembly in TAG carried out by different transferases (PDCT, PDAT, LPCAT and others) and favouring the synthesis of PE from DAG. It is also remarkable that a similar phenotype has not been depicted in Arabidopsis to date.

## Conclusions

Lipoyl synthases are essential enzymes in LA biosynthesis, a key co-factor of several enzyme complexes involved in central metabolism, including PDH regulation. Two sunflower lipoyl synthases were cloned and studied here, containing both the highly conserved RS and auxiliary [4S-4Fe] clusters. Heterologous expression of these enzymes in *E. coli* did not significant affect bacterial fatty acid composition, yet both enzymes complemented the lipoyl synthase activity in a defective mutant *E. coli*. Arabidopsis seeds overexpressing these enzymes do not experience significant differences in their total fatty acid composition but an unexpected remodeling of their glycerolipid species. While this excess lipoyl synthase activity does not affect plastidial PDH lipoylation it may sequester SAM, which is also required for PC synthesis from DAG. Accordingly, the reduced availability of SAM could diminish the PC generated and lead to an accumulation of PE species and this unbalance content of PC and DAG affect the enzymes using these substrates (PDCT, PDAT or LPCAT) for the incorporation of fatty acids in TAG modifying their distribution.

## Supplementary information

Supplementary Table and figures.
