# Supplementary material for: Impact of sunflower (Helianthus annuus L.) plastidial lipoyl synthases genes expression in glycerolipids composition of transgenic Arabidopsis plants
Source: Sci Rep. 2020 Feb 28;10:3749. doi: 10.1038/s41598-020-60686-z (PMC7048873; doi:10.1038/s41598-020-60686-z)
Supplement: Supplementary file 1 — Supplementary Table and figures. [file 41598_2020_60686_MOESM1_ESM.docx]

**Title: “Impact of sunflower (*Helianthus annuus* L.) plastidial lipoyl synthases genes expression in glycerolipids composition of transgenic Arabidopsis plants”**

**Authors:** Martins-Noguerol, Raquel; Moreno-Pérez, Antonio Javier; Sebastien, Acket; Troncoso-Ponce, Manuel Adrián; Garcés, Rafael; Thomasset, Brigitte; Salas, Joaquín J.; Martínez-Force, Enrique.

**Supplementary Table S1.** PCR primer sequences used in this study.

| **Primer** | **Sequence (5´- 3´) ^a^** |
| --- | --- |
| HaLIP1p1-*Bam*HI-F | T**GGATCC**ATGCTTATTCAACAATTCAACAC |
| HaLIP1p1-*Hind*III-R | G**AAGCTT**TCACAAGTAACTTTTGTTCTTC |
| HaLIP1p1-B-*Bam*HI-F | T**GGATCC**AAGAAACCGGGTTGGCT |
| HaLIP1p2-*Sph*I-F | T**GCATGC**ATGATGATGATGTTCAGCAAC |
| HaLIP1p2-*Xba*I-F | G**TCTAGA**ATGATGATGATGTTCAGCAAC |
| HaLIP1p2-*Hind*III-R | G**AAGCTT**TCATTTTGATGTGTTTTTGGTC |
| HaLIP1p2-B-*Bam*HI-F | T**GGATCC**AAGAAACCGGGTTGGCT |
| HaLIP1p1qpcr-F | GTTACACCTGAGAAATTCG |
| HaLIP1p1qpcr-R | TTCTTCGACTTGACCAATG |
| HaLIP1p2qpcr-F | GATACGAGCAGAGTTTATC |
| HaLIP1p2-qpcr-R | GCAAATCATTCATGGCTTC |
| HaActin-qpcr-F4 | GCTAACAGGAAAAGATGACT |
| HaActin-qpcr-R4 | ACTGGCATAAAGAGAAAGCACG |
| CAMV35S-F | CGTAAGGGATGACGCACAAT |
| pBIN19-R | CACACAGGAAACAGCTATGACC |

**^a^** Restriction sites are indicated in bold


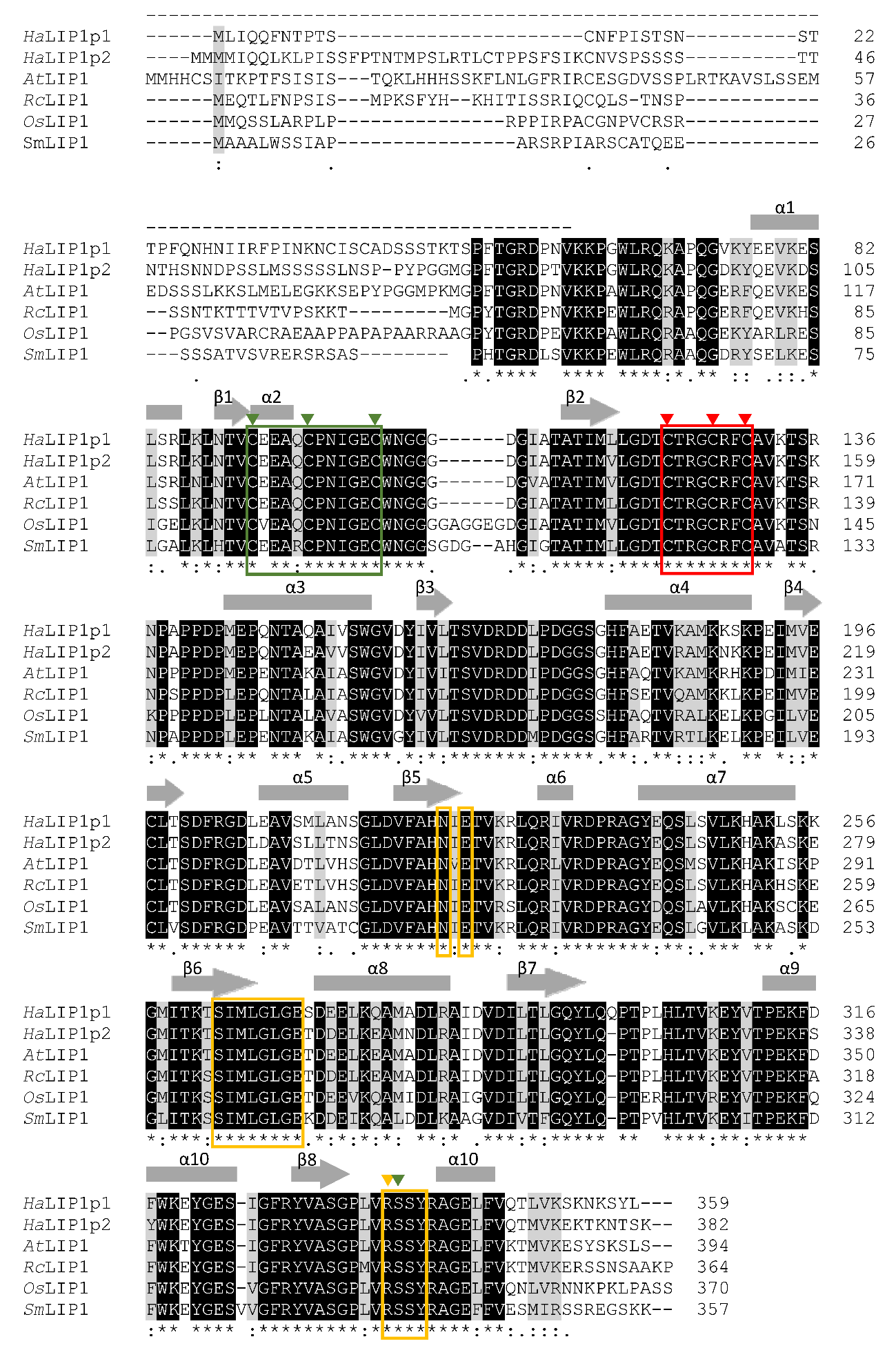


Supplementary Figure S1. Alignment of the amino acid sequences of plastidial *Helianthus annuus* lipoyl synthases (*Ha*LIP1p1 and *Ha*LIP1p2) with the closely related sequences from *Arabidopsis thaliana* (*At*LIP1), *Ricinus communis* (*Rc*LIP1), *Oryza sativa* (*Os*LIP1) and *Selaginella moellendorffii* (*Sm*LIP1). Identical residues are highlighted as black boxes and highly conserved residues as dark grey boxes. The secondary structure elements are represented by cylinders (α-helix) and arrows (β-strand). Putative N-terminal plastidial transit peptide is indicated with a dashed line. [4Fe-4S] clusters are indicated in red box (RS cluster involved in SAM cleavage) and green box (auxiliary cluster). C-terminal R(S/T)S conserved motif is boxed in blue with the Ser ligand to auxiliary cluster indicated with a green arrow. The typical RS motifs for recognizing s-adenosyl-methionine, SAM, are marked in orange boxes (both Asn and Glu of Ribose Motif and the “GXIXGXXE” motif) and with orange arrow (R).


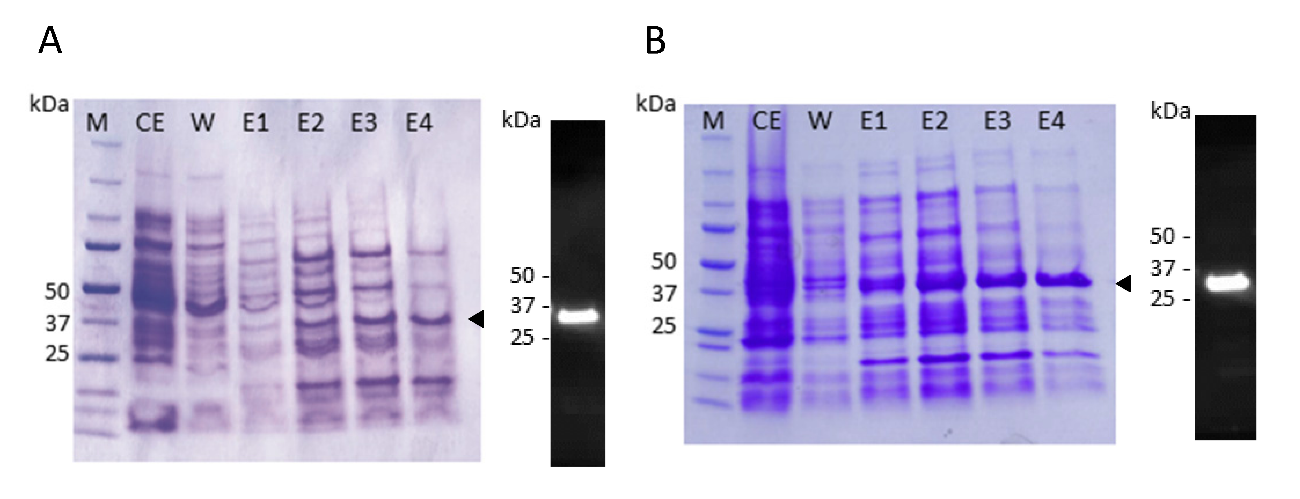


Supplementary Figure S2. Purification of recombinant mature *Ha*LIP1p1 (A) and *Ha*LIP1p2 (B) from *E. coli* extract. M, protein standard markers; CE, crude extract; W, wash sample; E1-E4, eluted fractions with different imidazole concentrations. Western Blots with AntiHis Peroxidase Monoclonal Antibodies of purified proteins from E4 fractions are shown next to the gels. Once removed the signal peptides, both recombinant proteins have an expected molecular weight of 33.1 kDa.


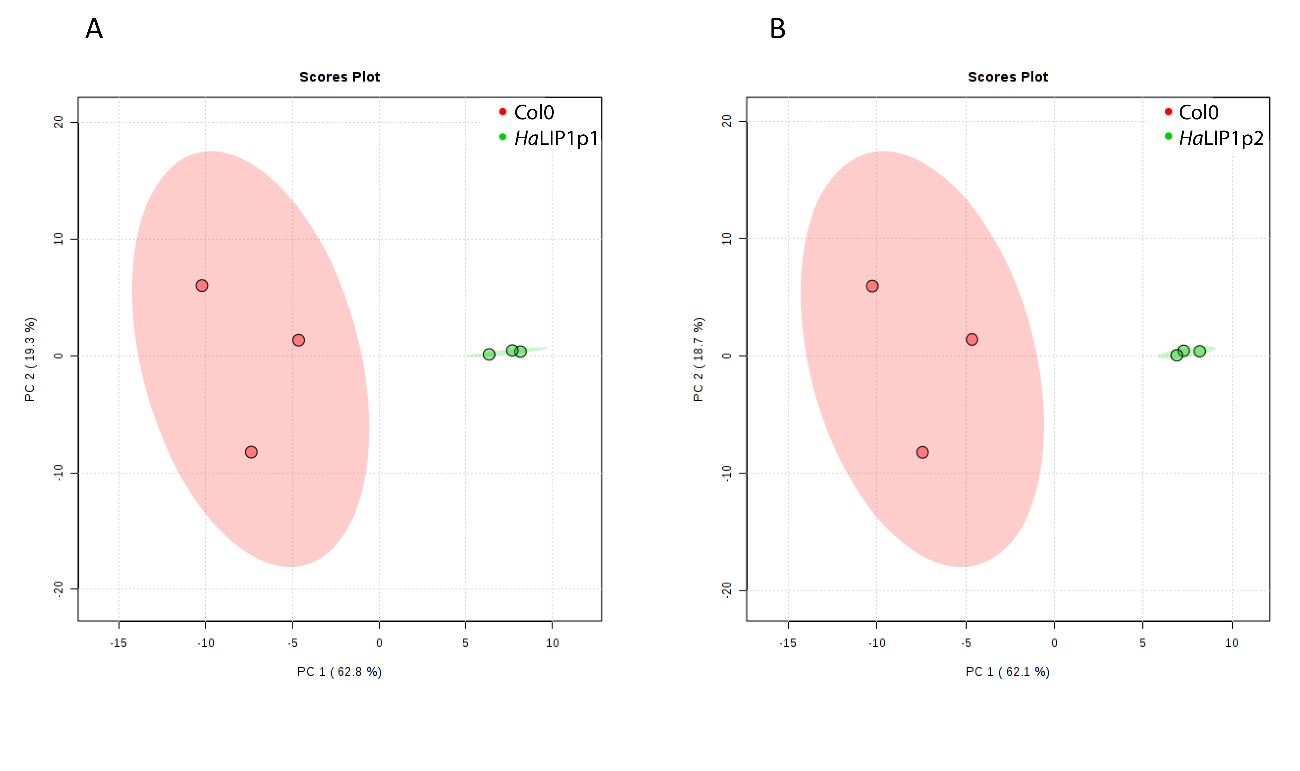


Supplementary Figure S3. Scores plots between the selected principal component analysis comparing Col0 plants lipid species with transgenic plants expressing *HaLIP1p1* (A) or *HaLIP1p2* (B). The explained variances are shown in brackets.


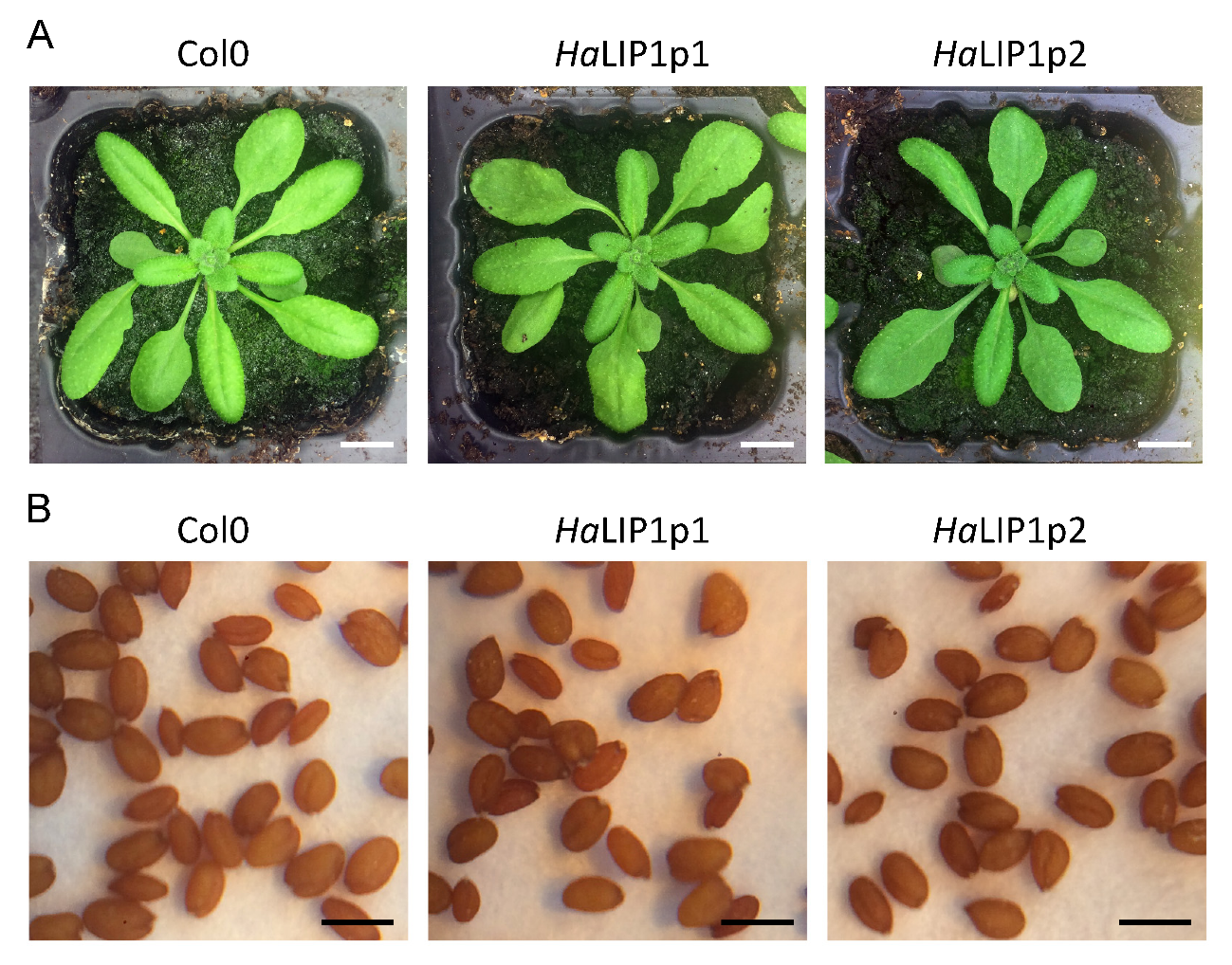


Supplementary Figure S4. (A) Pictures of Arabidopsis control plant (Col0) and plants transformed with *Ha*LIP1p1 and *Ha*LIP1p2 at 26 days after germination. (B) Mature seeds harvested from control Arabidopsis plant (Col0) and plants transformed with *Ha*LIP1p1 and *Ha*LIP1p2. Bars corresponded to 1 cm in (A) and 0.5 mm in (B).
